# Combination of β-Aminobutyric Acid and Ca^2+^ Alleviates Chilling Stress in Tobacco (*Nicotiana tabacum* L.)

**DOI:** 10.3389/fpls.2020.00556

**Published:** 2020-05-13

**Authors:** Xiao-Han Ma, Jia-Yang Xu, Dan Han, Wu-Xing Huang, Bing-Jun Dang, Wei Jia, Zi-Cheng Xu

**Affiliations:** ^1^College of Tobacco Science, Henan Agricultural University, Zhengzhou, China; ^2^College of Agronomy and Biotechnology, China Agricultural University, Beijing, China

**Keywords:** chilling stress, β-aminobutyric acid, calcium ion, oxidative stress, membrane lipid damage

## Abstract

Chilling is a major abiotic factor limiting the growth, development, and productivity of plants. β-aminobutyric acid (BABA), a new environmentally friendly agent, is widely used to induce plant resistance to biotic and abiotic stress. Calcium, as a signaling substance, participates in various physiological activities in cells and plays a positive role in plant defense against cold conditions. In this study, we used tobacco as a model plant to determine whether BABA could alleviate chilling stress and further to explore the relationship between BABA and Ca^2+^. The results showed that 0.2 mM BABA significantly reduced the damage to tobacco seedlings from chilling stress, as evidenced by an increase in photosynthetic pigments, the maintenance of cell structure, and upregulated expression of *NtLDC1*, *NtERD10B*, and *NtERD10D*. Furthermore, 0.2 mM BABA combined with 10 mM Ca^2+^ increased the fresh and dry weights of both roots and shoots markedly. Compared to that with single BABA treatment, adding Ca^2+^ reduced cold injury to the plant cell membrane, decreased ROS production, and increased antioxidant enzyme activities and antioxidant contents. The combination of BABA and Ca^2+^ also improved abscisic acid and auxin contents in tobacco seedlings under chilling stress, whereas ethylene glycol-bis (β-aminoethylether)-*N,N,N*′,*N*′-tetraacetic acid (EGTA) reversed the effects of BABA. These findings suggested that BABA enhances the cold tolerance of tobacco and is closely related to the state of Ca^2+^ signaling.

## Introduction

Global climate change has led to a worldwide increase in the frequency of low temperature periods in recent years (Augspurger, 2013; Liu et al., 2014). Chilling stress is an important environmental factor limiting plant growth and development, and ultimately causes damage to the whole plant (Liu et al., 2019), which further threatens both the productivity and quality of crops. Chilling during the reproductive period of rice causes spikelet degradation and sterility (Wang et al., 2013). Moreover, low temperatures in spring reduce wheat yield by 30–50% (Zhang et al., 2011). Further, the exposure of maize seedlings to low temperature induces leaf chlorosis and necrosis and inhibits plant growth (Moradtalab et al., 2018). Cold stress also results in a reduction in cotton and soybean yield (Dong et al., 2006; Kidokoro et al., 2015). However, the cold tolerance of plants is an intricate trait that is involved in different physiological and biochemical processes and modulating this characteristic poses a formidable challenge to conventional plant breeding (Zhang et al., 2019). Therefore, ways to potentiate plant cold tolerance have become an important issue, and related measures must be developed.

Beta-aminobutyric acid (BABA), widely described as a defense-priming molecule, confers plant resistance to a broad spectrum of biotic and abiotic stresses including microbial pathogens, arthropods, salt, and heat shock (Maymoune et al., 2015; Mostek et al., 2016; Yao et al., 2019). Prior research confirmed that BABA helps soybean to combat Cd stress and barley to resist salt stress. Based on ABA-dependent defense mechanisms, BABA induces higher tolerance to drought and salinity in *Arabidopsis* and *Vigna radiata* (Jakab et al., 2005; Jisha and Puthur, 2016). It has also been reported to enhance drought resistance in maize by increasing mRNA and protein expression (Shaw et al., 2016). In addition, BABA changes the response of leaf antioxidants to UV-B, and Mátai et al. (2019) determined that it might be a new stress hormone.

Calcium ion (Ca^2+^), which is known as a ubiquitous second messenger, plays a vital role in plant growth and development (Yang et al., 2016). Specifically, it participates in physiological regulation (Shao et al., 2008), which was also shown to improve plant resistance to various adversities including salt, drought, and chilling (Wood et al., 2000; Cramer et al., 2006; Jeon and Kim, 2013; Jia et al., 2017). EGTA is Ca^2+^ chelator that reduces endogenous calcium ions (Zhou and Guo, 2009). Cramer et al. (2006) found that Ca^2+^ alleviates salt stress by reducing the Na^+^ level in barley. Further, exogenous Ca^2+^ application was reported to enhance plant drought resistance by coupling extracellular stimuli with intracellular responses (Shao et al., 2008). Especially, previous studies have documented that Ca^2+^ can mitigate cold-induced damage to plants (Kitagawa and Yoshizaki, 1998; Wood et al., 2000). The Ca^2+^ signaling pathway is also an important part of the plant defense system that responds to low temperature (Jeon and Kim, 2013). Low temperature stress leads to an increase in free Ca^2+^ concentrations through Ca^2+^ transmembrane influx, opening the calcium ion channel and therefore causing Ca^2+^ to flow into the cytoplasm (Jia et al., 2017). Frequently implemented, Ca^2+^ alleviates hypothermic damage, which might be involved in improving osmotic stress through signaling processes and the restoration of cell membrane integrity (Zheng et al., 2014).

β-aminobutyric acid is widely accepted as a new environmentally friendly agent (Li et al., 2019). Although many studies have demonstrated that it induces tolerance to different stresses in plants, little information has been obtained regarding its effect on plant cold tolerance. Furthermore, whether a combination of BABA and Ca^2+^ could result in a better mitigative effect with respect to plant resistance to chilling is also not clear. We hypothesized that BABA can improve plant resistance to chilling stress and that the combined effect of BABA and Ca^2+^ would be better. In this study, tobacco served as a model plant that is susceptible to chilling stress, and this was used to (1) investigate the ameliorative effect of BABA on chilling stress and the related mechanisms and (2) explore the joint action of BABA and Ca^2+^ on plant cold tolerance. The results of this study will not only expand the application of BABA for plants in harsh environments but could also provide an eco-friendly method to promote plant resistance to chilling stress.

## Materials and Methods

### Plant Material, Treatment, and Sample Collection

We used the tobacco variety “Yuyan No. 10” as an experimental plant material, using a hydroponic culture method. Tobacco seeds were sterilized with 10% hydrogen peroxide (H_2_O_2_), rinsed thoroughly with distilled water, and germinated at 28°C in the dark in an I-shaped square seedling sponge, supplied with Hoagland’s solution. The germinated seeds were transferred to the vermiculite for subsequent growth. Upon reaching the four-leaf stage, the seedlings were transferred to 5-L plastic pots with Hoagland nutrient solution. The solution was continuously aerated and renewed once every 3 days.

Experiment I: At the six-leaf stage, tobacco plants were sprayed one time per day with BABA (0, 0.1, 0.2, 0.5, and 1.0 mM) for 3 days. Then, plants were subjected to cold stress (8/4°C) for 5 days and control plants were grown at normal temperature (28/18°C). The specification of the cold stress temperature and days of stress are indicated in Supplementary Material. After 5 days, two true leaves were selected to determine the physiological and biochemical indexes to ensure the optimal BABA concentration for tobacco plant growth. Each treatment was replicated three times.

Experiment II: We tested the interactive effect of BABA and Ca^2+^ on tobacco. Seedlings were exposed to 0.2 mM BABA, 10 mM Ca^2+^ solution, BABA + Ca^2+^, and BABA + 3 mM EGTA for 5 days in the cold. EGTA is a calcium ion chelating agent that reduces Ca^2+^ in plants after application. On days 0, 3, and 5 of treatment, we collected two true leaves to determine the physiological and biochemical indexes. Three biological replicates were performed for each experiment.

### Morphological Indicators and Biomass

Morphological indicators were estimated by measuring the root length, leaf length, leaf width, and leaf area of the same leaf position of the plant. Biomass determination included fresh weight and dry weight. First, the fresh weight of the seedlings was determined. Dry weight was measured by oven-drying leaves at 105°C for 30 min and dried at 70°C for 48 h.

### Electrolyte Leakage Assay and Determination of Antioxidant Enzyme Activities

Electrolyte leakage was measured using a Model DDS-11A conductivity meter (Leici, Shanghai). Leaves were collected at 0, 3, and 5 days following cold treatment for the determination of electrolyte leakage. Electrolyte leakage was measured following the method described by Köhle et al. (1985). Leaves were cut into 0.5 g and immersed in 25 mL of deionized water in a test tube for 24 h at room temperature, and the conductivities of the obtained solutions were determined (R1). Then, the tubes were boiled for 30 min and cooled to room temperature, and the conductivity was determined again. The measured data were recorded as R2. The rate of electrolyte leakage was calculated using the following equation: electrolyte leakage (%) = (R1/R2) × 100%. Assays of catalase activity (CAT), peroxidase activity (POD), and superoxide dismutase activity (SOD) enzymes were performed in accordance with the methods described by Zhang and Kirkham (1996). Specifically, 0.5 g of fresh leaf segments were homogenized in 5 mL of 50 mM potassium phosphate buffer (pH 7.8) containing 0.1 mM EDTA and 1% PVP in an ice bath. The homogenate was centrifuged at 10,000 × *g* for 20 min at 4°C and the supernatant was used for enzyme assays. For the assay of CAT activity, the 3 mL reaction mixture contained 50 mM phosphate buffer (pH 7.0), 15 mM H_2_O_2_, and 0.1 mL enzyme extract. The decomposition of H_2_O_2_ was followed by the decline in absorbance at 240 nm for 1 min. POD activity was measured using a guaiacol substrate. The reaction mixture contained 50 μL of 20 mM guaiacol, 2.83 mL of 10 mM phosphate buffer (pH 7.0), and 0.1 mL enzyme extract, with the increase in absorbance at 470 nm for measured 1 min. Total SOD activity was determined by measuring its ability to inhibit the photochemical reduction of nitro blue tetrazolium (NBT). The 3 mL reaction mixture contained 63 μM NBT, 1.3 μM riboflavin, 13 mM methionine, 0.1 mM EDTA, 50 mM phosphate buffer (pH 7.8), 20 μL of enzyme extract and, 20 μM of riboflavin. The absorbance of the solution was measured at 560 nm.

### Non-enzymatic Antioxidant Assays

Glutathione (GSH) concentration was measured by the method of Law et al. (1983). The crude extract was obtained by homogenizing 0.5 g of fresh leaves in 5 mL 5% (w/v) TCA solution, which was then centrifuged at 12,000 × *g* for 20 min at 4°C. The reaction mixture consisted of 0.5 mL water, 2 mL of 0.2 mM phosphate buffer solution (pH 7.0), 0.5 mL supernatant, and 0.1 mL DTNB. The content of GSH was calculated by measuring the absorbance at 412 nm. Ascorbic acid (AsA) concentration was measured by the method of Law et al. (1983). The crude extract was obtained by homogenizing 0.5 g of fresh leaves in 5 mL 5% (w/v) TCA solution, which was then centrifuged at 12,000 × *g* for 20 min at 4°C. The determination was based on the reduction of Fe^3+^ to Fe^2+^ by AsA, and Fe^2+^ was quantified spectrophotometry at 534 nm at 30°C.

### Determination of Malondialdehyde (MDA) Content

Malondialdehyde concentration was measured according to the method of Hodges et al. (1999). The supernatant was obtained by homogenizing 0.5 g of fresh leaves in 5 mL of distilled water, which was then added to 5 mL 0.5% thiobarbituric acid, extracted in a boiling water bath for 20 min, and centrifuged. The content of MDA was calculated by measuring the absorbance at 450, 532, and 600 nm.

### Determination of ROS Accumulation

H_2_O_2_ content was measured as described by Jana and Choudhuri (1982). For this assay, 0.5 g of fresh leaves was homogenized in 5 mL of 50 mM phosphate buffer and centrifuged. The analytical mixture that contained 1 mL of 0.1% titanium sulfate and 3 mL of the supernatant was centrifuged at 7,000 × *g* for 15 min. The content of H_2_O_2_ was calculated by measuring the absorbance at 410 nm.

Superoxide (O_2_^–^) content was detected using the method of Jabs et al. (1996). In addition, 0.5 g of fresh leaves was homogenized in 5 mL of 50 mM phosphate buffer and centrifuged at 12,000 × *g* for 10 min at 4°C. The reaction mixture consisted of 1 mL supernatant, 1 mL 50 mM phosphate buffer, 1 mL 17 mM p-aminobenzenesulfonic acid, and 1 mL 7 mM α-naphthylamine. After 20 min of color development at 25°C, the content of O_2_^–^ was calculated by measuring the absorbance at 530 nm.

### Photosynthetic Pigments

The determination of chlorophyll *a* (Chl *a*), chlorophyll *b* (Chl *b*), and carotenoid (Car) was performed following the method proposed by Green and Durnford (1996). Leaf samples (0.2 g) were extracted in 10 mL of 80% acetone for 24 h, and the absorbance of the extract was measured at 665, 649, and 470 nm.

### Ultrastructure

Leaves containing no veins were cut into small pieces of 1 mm × 2 mm for fixation, dehydration, and embedding. The leaves were fixed for 2 h at 4°C in 2.5% (v/v) glutaraldehyde in 0.05 M phosphate buffer (pH 7.2) and then with 2% (w/v) osmium tetroxide in phosphate buffer. The samples were embedded in epoxy resin after dehydration in an ethanol gradient (30, 50, 70, 90, and 100%, twice each for 30 min). The sheets were cut with a Leica EMUC6 ultrathin slicer and stained with lead citrate and uranyl acetate. Finally, a transmission electron microscope was used to observe the chloroplast ultrastructure and take photographs.

### Hormone Content

The contents of abscisic acid (ABA) and auxin (IAA) were determined by the method of Guinn et al. (1986) and Veselov et al. (2010). First, 0.1 g of fresh leaves was homogenized in 80% ethanol and centrifuged at 8,000 × *g* for 10 min at 4°C. The supernatant was decolorized by adding 0.5 mL of petroleum ether three times. The nitrogen blowing device was blown to a liquid level of 0.5 mL or less. The mobile phase was brought to a volume of 0.5 mL to be tested. It was acidified to pH 2.5 with HCl and partitioned twice with diethyl ether. ABA and IAA were transferred from the organic phase to 1% sodium bicarbonate and extracted again with diethyl ether. Finally, ABA and IAA were immunoassayed with diazomethane methylation.

### Total RNA Extraction and Real-Time PCR Analysis

Total RNA was extracted from tobacco leaf tissue using the Plant Total RNA Isolation Kit QuantiFast^®^ SYBR^®^ Green PCR Kit (Qiagen, Germany) according to the manufacturer’s specifications. The yield of RNA was determined using a NanoDrop 2000 spectrophotometer (Thermo Scientific, United States), and the integrity was evaluated using agarose gel electrophoresis staining with ethidium bromide. Quantification was performed with a two-step reaction process comprising reverse transcription (RT) and PCR. Each RT reaction had two steps. The first step included 0.5 μg RNA (OD 260/280, 2.13–2.16; OD 260/230, 1.80–2.13), 2 μL of 4× gDNA wiper Mix, and nuclease-free H_2_O to 8 μL. Reactions were performed in a GeneAmp^®^ PCR System 9700 (Applied Biosystems, United States) for 2 min at 42°C. In the second step, 2 μL of 5× HiScript II Q RT SuperMix IIa was added. Reactions were performed in a GeneAmp^®^ PCR System 9700 (Applied Biosystems, United States) for 10 min at 25°C, 30 min at 50°C, and 5 min at 85°C. The 10-μL RT reaction mix was then diluted 10-fold in nuclease-free water and stored at −20°C.

Real-time PCR was performed using a LightCycler^®^ 480 II Real-time PCR Instrument (Roche, Swiss) with 10 μL of PCR reaction mixture that included 1 μL of cDNA, 5 μL of 2 × QuantiFast^®^ SYBR^®^ Green PCR Master Mix (Qiagen, Germany), 0.2 μL of forward primer, 0.2 μL of reverse primer, and 3.6 μL of nuclease-free water. Reactions were incubated in a 384-well optical plate (Roche, Swiss) at 95°C for 5 min, followed by 40 cycles of 95°C for 10 s and 60°C for 30 s. Each qRT-PCR reaction was performed with three biological replicates and three technical replicates. At the end of the PCR cycles, melting curve analysis was performed to validate the specific generation of the expected PCR product. All tobacco gene primer sequences were designed in the laboratory and synthesized by Generay Biotech (Generay, PRC) based on the mRNA sequences obtained from the NCBI database^1^ as follows: for amplification, primer sequences were GGAGGATGATGGACAAGGC (forward) and GTCTGATCGTCTTTGTGACC (reverse) for *NtERD10B* (AB049336.1), CGAGGGAAGAAGAGAAGGC (forward) and GTTTCCTCGTATTTCTCCACTG (reverse) for *NtERD10D* (AB049338.1), CAGCAAAGGAGCTGGTCA (forward) and CAACTTGGAAGCAACTCG (reverse) for *NtLDC1* (KU507075.1), and CCCCTCACCACAGAGTCTGC (forward) and AAGGGTGTTGTTGTCCTCAATCTT (reverse) for *L25* (L18908.1). The expression levels of mRNA were normalized to those of *L25* and were calculated using the 2^–ΔΔCt^ method (Livak and Schmittgen, 2001).

### Statistical Analysis

The data for each measurement represent the means of three replicates. The data were analyzed using an analysis of variance (ANOVA) and Duncan multiple comparisons to detect differences between treatments. Differences were considered significant at *P* < 0.05. All statistical analyses were performed using SPSS 19.0.

## Results

### BABA Increases Tobacco Morphological Parameters Under Chilling Stress

The effects of BABA on tobacco morphological index parameters are shown in Table 1. Low temperature significantly decreased root length, leaf length, leaf width, and leaf area. However, the exogenous application of BABA promoted the growth of tobacco plants under cold stress to varying degrees at different concentrations. Compared to those with cold treatment, 0.2 mM BABA increased root length, leaf length, leaf width, and leaf area, by 84.62, 20.85, 3.97, and 25.76%, respectively (Table 1). In addition, 0.5 mM BABA resulted in the maximum leaf width and leaf area, among the four BABA concentrations tested, and improved these parameters by 36.38 and 51.70%, respectively, compared to those with cold treatment.

**TABLE 1:** Morphological parameters of tobacco treated with BABA under cold stress.

Treatment	Root length (cm)	Leaf length (cm)	Leaf width (cm)	Leaf area (cm^2^)
Control	24.83 ± 1.29 a	24.13 ± 0.57 a	11.40 ± 0.44 b	174.50 ± 5.30 a
Cold	13.00 ± 1.68 d	17.27 ± 0.35 d	9.07 ± 0.21 c	99.30 ± 0.47 d
0.1 mM BABA	17.33 ± 0.50 c	17.47 ± 0.47 d	9.53 ± 0.31 c	105.68 ± 5.18 d
0.2 mM BABA	24.00 ± 1.97 a	20.87 ± 0.31 b	9.43 ± 0.32 c	124.88 ± 4.01 c
0.5 mM BABA	20.69 ± 0.61 bc	19.20 ± 0.70 c	12.37 ± 0.25 a	150.64 ± 5.59 b
1 mM BABA	15.70 ± 0.96 c	17.33 ± 0.45 d	9.10 ± 0.20 c	100.06 ± 2.56 d

*The data are means ± SE, n = 3. Different letters indicate a statistical difference at P < 0.05 among samples according to Duncan’s tests.*

### BABA Improves Photosynthetic Pigment Content of Tobacco Plants Exposed to Chilling Stress

The Chl *a*, Chl *b*, and Car contents of tobacco plants were significantly decreased with cold treatment by 29.59, 34.02, and 28.91%, respectively, from control levels (Table 2). The total amount of leaf pigments after treatment with exogenous BABA was significantly increased, reaching a peak with 0.2 mM BABA treatment, which was 39.78% higher than that observed upon cold treatment. Car levels exhibited the same trend and were elevated by 29.12% with 0.2 mM BABA as compared to those after cold treatment. These results indicated that 0.2 mM BABA significantly relieves the decrease in tobacco photosynthetic pigment content caused by cold stress.

**TABLE 2:** Effect of BABA on photosynthetic pigment content in tobacco seedlings under cold stress.

Treatment	Chl *a* (mg g^–1^ FW)	Chl *b* (mg g^–1^ FW)	Car (mg g^–1^ FW)	Chl (*a* + *b*) (mg g^–1^ FW)
Control	15.48 ± 0.10 a	5.82 ± 0.27 a	2.56 ± 0.14 a	21.30 ± 0.19 a
Cold	10.99 ± 0.35 d	3.84 ± 0.51 d	1.82 ± 0.08 b	14.83 ± 0.18 c
0.1 mM BABA	12.72 ± 0.75 c	4.92 ± 0.35 ab	2.25 ± 0.14 ab	17.64 ± 0.75 b
0.2 mM BABA	14.91 ± 0.04 a	5.82 ± 0.44 a	2.35 ± 0.13 a	20.73 ± 0.46 a
0.5 mM BABA	13.62 ± 0.22 b	4.50 ± 0.18 bc	2.34 ± 0.13 ab	18.12 ± 0.23 b
1 mM BABA	11.48 ± 0.44 d	3.75 ± 0.99 d	2.28 ± 0.47 ab	15.23 ± 0.57 c

*Data represent means ± SE (n = 3). Values in the same column denoted by different letters are significantly different at the 0.05 level (Duncan’s test).*

### BABA Maintains the Ultrastructure of Tobacco Plants

**FIGURE 1:**
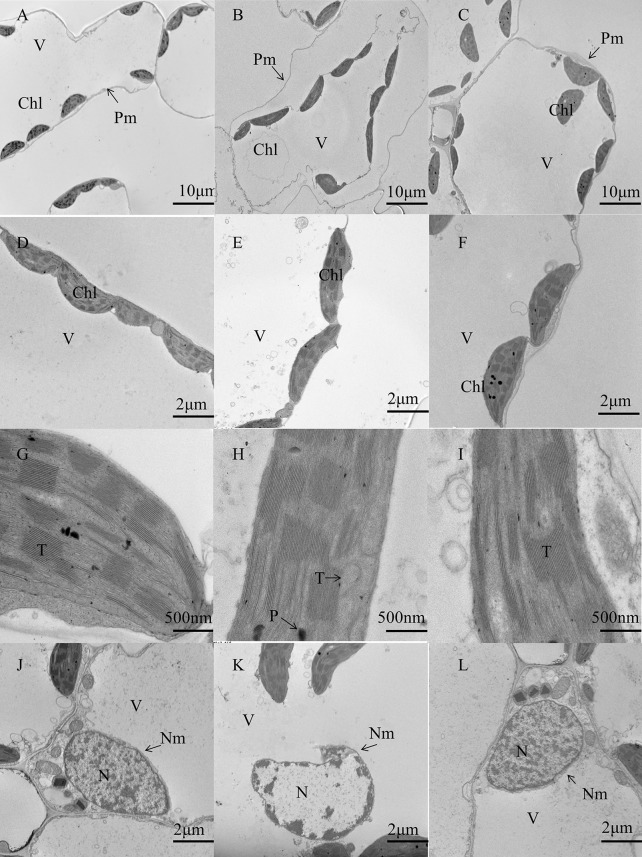
Effect of BABA on the chloroplast ultrastructure in tobacco under cold stress. Ultrastructural changes with control treatment **(A,D,G,J)**, cold treatment **(B,E,H,K)**, and cold + 0.2 mM BABA treatment **(C,F,I,L)**. V, vacuolar; Chl, chloroplast; T, thylakoid; N, nuclear; Nm, nuclear membrane; Pm, plasma membrane; p, plastoglobule.

Based on these results, we chose three treatments to further investigate leaf ultrastructure. Compared to that observed in control conditions (Figures 1A,D), the leaves exposed to cold treatment showed severe wall separation of the chloroplast (Figures 1B,E) and the partial ablation of thylakoid layers, as well as the existence of some osmiophilic drops (Figure 1H). The internal structure of the nucleus was disordered under cold treatment (Figure 1K), whereas most of the organelles of BABA-treated leaves attached to the cell wall (Figures 1C,F). The thylakoid layers and nuclear membrane of plants of the control and BABA treatment groups were normally and clearly visible (Figures 1G,I,J,L). These results indicate that low temperature destroys the chloroplast and nucleus, whereas BABA alleviates cell damage due to low temperature stress.

### BABA Upregulates Stress-Related Gene Expression

**FIGURE 2:**
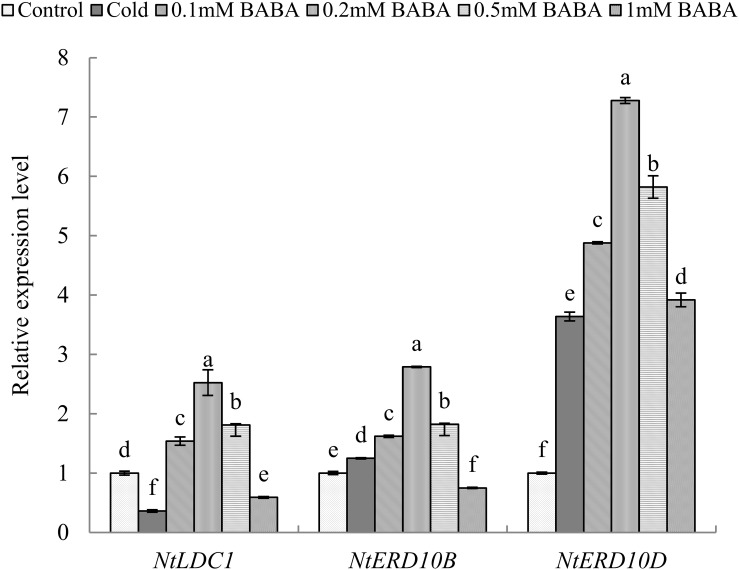
Relative expression of genes in response to cold stress in tobacco. Gene expression was analyzed by qRT-PCR. Seedlings were pre-treated with β-aminobutyric acid (BABA) for 3 days prior to cold stress. The *L25* gene was used as an internal control. The gene level in the control treatment group was given an arbitrary value of 1. Gene expression data were normalized to levels in the control group. Data are representative of three biological replicate experiments. Different letters indicate a significant difference between means (*P* < 0.05).

We next quantified the expression levels of three genes that are closely related to chilling stress in the six treatments. Compared to that with control treatment, the *NtLDC1* gene was downregulated significantly with cold treatment, whereas *NtERD10B* and *NtERD10D* genes were upregulated. Upon BABA (0.1, 0.2, and 0.5 mM) treatment, the *NtLDC1* gene was significantly upregulated by 1. 54-, 2. 52-, and 1.81-fold relative to control levels, respectively. However, 1 mM BABA reduced expression by 40.95% compared to that with control treatment. *NtERD10B* gene expression exhibited the same trend as *NtLDC1* following BABA treatment, showing upregulation at concentrations of 0.1, 0.2, and 0.5 mM and downregulation with 1 mM BABA (Figure 2). We could speculate that excess BABA might aggravate the adverse effects on tobacco seedlings exposed to cold stress, which is consistent with the results of the physiological and biochemical analyses.

### Combining BABA With Ca^2+^ Facilitates Tobacco Growth Under Cold Stress

As shown in Table 3, the fresh and dry weights of the BABA, Ca^2+^, and BABA + Ca^2+^ groups were increased significantly compared to those with cold treatment. Especially, BABA + Ca^2+^ treatment resulted in maximum fresh and dry weights of roots and shoots at the same time, which were increased by 94.99 and 93.28%, as well as 30.84 and 100.04% relative to those with cold treatment, respectively. However, no significant differences in fresh and dry weight were detected between BABA + EGTA treatment and cold treatment groups. Furthermore, the fresh weight of roots and shoots under BABA + Ca^2+^ treatment was increased by 16.88 and 22.41%, and the dry weight was improved by 15.10 and 25.18% from that of the single BABA treatment, respectively. Meanwhile, BABA + Ca^2+^ also contributed to the elevated fresh weight of roots (5.51%) and shoots (6.95%), and dry weight of roots (4.21%) and shoots (8.52%), separately, compared with the Ca^2+^ treatment (Table 3). These results suggest that the application of BABA, Ca^2+^, and BABA + Ca^2+^ in a low temperature environment can significantly relieve the damage to tobacco seedlings caused by chilling injury and that BABA + Ca^2+^ results in the best mitigative effect. In contrast, the biomass with BABA + EGTA treatment was significantly reduced.

**TABLE 3:** Effects of BABA, Ca^2+^, BABA + Ca^2+^, and BABA + EGTA on the growth of tobacco seedlings under cold stress.

Treatment	Fresh weight (g)		Dry weight (g)	
	Shoot (g)	Root (g)	Shoot (g)	Root (g)
Cold	11.17 ± 1.02 cd	1.27 ± 0.13 c	1.51 ± 0.10 b	0.11 ± 0.01 b
BABA	18.64 ± 0.80 b	2.01 ± 0.03 b	1.88 ± 0.05 a	0.21 ± 0.01 a
Ca^2+^	18.93 ± 0.31 b	1.97 ± 0.06 b	1.90 ± 0.05 a	0.21 ± 0.01 a
BABA + Ca^2+^	21.79 ± 1.52 a	2.46 ± 0.16 a	1.98 ± 0.06 a	0.22 ± 0.03 a
BABA + EGTA	12.52 ± 0.60 c	1.10 ± 0.12 c	1.44 ± 0.03 b	0.12 ± 0.05 b

*The data are means ± SE, n = 3. Means followed by the same letter were not significantly different according to Duncan’s test significant difference (P < 0.05).*

### Joint Effect of BABA and Ca^2+^ on Reducing Electrolyte Leakage and MDA Content in Tobacco

Measurements of electrolyte leakage in leaf tissue are commonly used to assess the integrity of plant cell membranes. The MDA level is also a general marker of oxidative damage to lipid membranes, and there is a certain correlation between the two indicators. There were no significant differences in electrolyte leakage and MDA levels among treatments and before treatment with chilling stress. However, with time, electrolyte leakage and MDA contents in plant leaves increased sharply. Compared to that at day 0 of stress, electrolyte leakage after 3 and 5 days of chilling stress was increased by 1.11- and 2.65-fold, respectively. Analogously, elevated MDA levels at the 3rd and 5th days clearly increased, by 28.02- and 60.21-fold, respectively, as compared to those at day 0 of stress.

**FIGURE 3:**
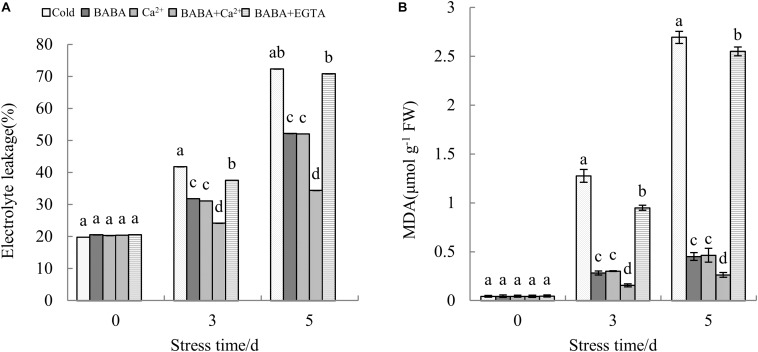
Effects of BABA, Ca^2+^, BABA + Ca^2+^, and BABA + EGTA on electrolyte leakage **(A)** and MDA **(B)** of tobacco under cold stress. Data are expressed as mean value ± SE, *n* = 3. Mean values in columns with different letters are significantly different at the 0.05 level according to Duncan’s test.

After being treated with chilling stress for 3 days, electrolyte leakage was 24.14% under BABA + Ca^2+^ treatment, which was the minimum value among the five treatments. However, no significant difference in electrolyte leakage was detected between BABA + EGTA and cold treatment groups. Further, electrolyte leakage after cold treatment and BABA + EGTA treatment was 2.10- and 2.06-fold greater compared to that with BABA + Ca^2+^ treatment after 5 days of chilling stress (Figure 3A). In accordance with electrolyte permeation, BABA + Ca^2+^ treatment also resulted in the lowest content over time (Figure 3B). These results might indicate that the combined effects of BABA and Ca^2+^ could alleviate chilling injury by reducing the increase in electrolyte permeation and MDA, ultimately protecting the cell membrane structure from increased lipid membrane permeability. However, BABA-induced chilling resistance was reversed by EGTA.

### Effects of Combined BABA and Ca^2+^ on Reducing ROS Accumulation and Improving Antioxidant Contents in Tobacco Leaves

**FIGURE 4:**
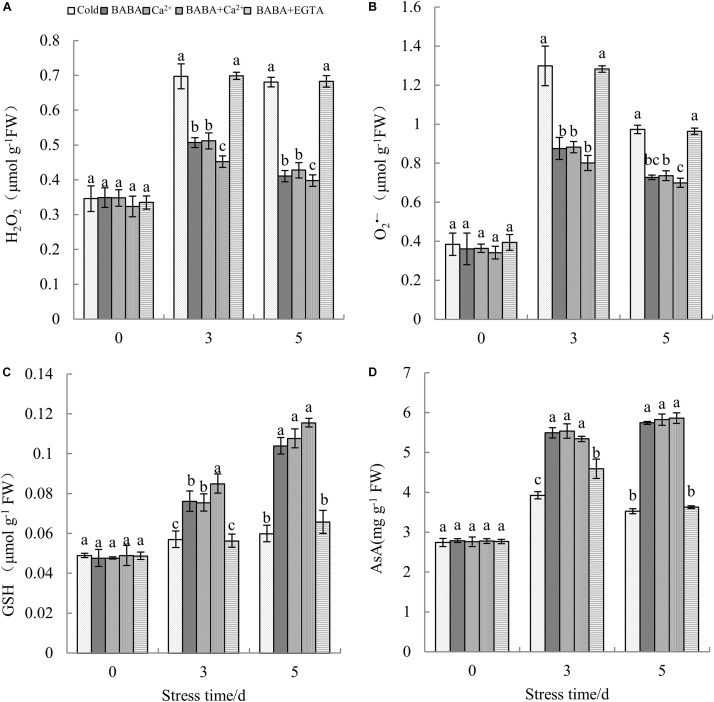
Effects of BABA, Ca^2+^, BABA + Ca^2+^, and BABA + EGTA on H_2_O_2_ **(A)**, O_2_^–^ **(B)**, GSH **(C)**, and AsA **(D)** of tobacco under cold stress. Data represent means ± SE (*n* = 3). Different letters denote significant differences according to Duncan’s test (*P* < 0.05).

ROS content reflects the level of peroxidation, and high ROS levels lead to serious membrane damage. Upon exposure to cold conditions, H_2_O_2_ levels in tobacco plants increased sharply (Figure 4A). Compared to that on day 0 of stress, H_2_O_2_ levels on the 5th day were increased by 96.88%. O_2_^–^ content tended to increase first and then decrease during the 5 days of chilling treatment (Figure 4B). Both BABA and Ca^2+^ reduced the levels of ROS, whereas BABA + Ca^2+^ resulted in the minimum effects. In contrast, BABA + EGTA resulted in the accumulation of more H_2_O_2_ and O_2_^–^ in tobacco leaves.

Plants have evolved a sophisticated array of antioxidant systems. The contents of non-enzyme antioxidants including GSH and AsA represent the antioxidant capacity. There was no significant difference in non-enzymatic antioxidant content among the five treatments and before treatment with chilling stress. Apparently, chilling induced the accumulation of GSH and AsA (Figure 4). Furthermore, exogenous BABA and calcium ions increased the accumulation of antioxidants under cold stress. After being treated with chilling stress for 5 days, GSH and AsA content peaked with BABA + Ca^2+^ treatment, and these levels were 92.65 and 66.24%, as well as 75.59 and 61.62%, higher than those with cold treatment and BABA + EGTA treatment, respectively. It could thus be surmised that the combined effect of BABA and Ca^2+^ could enhance the non-enzymatic antioxidant system better.

### BABA Combined With Ca^2+^ Improves Antioxidant Enzyme Activities in Tobacco Leaves

**FIGURE 5:**
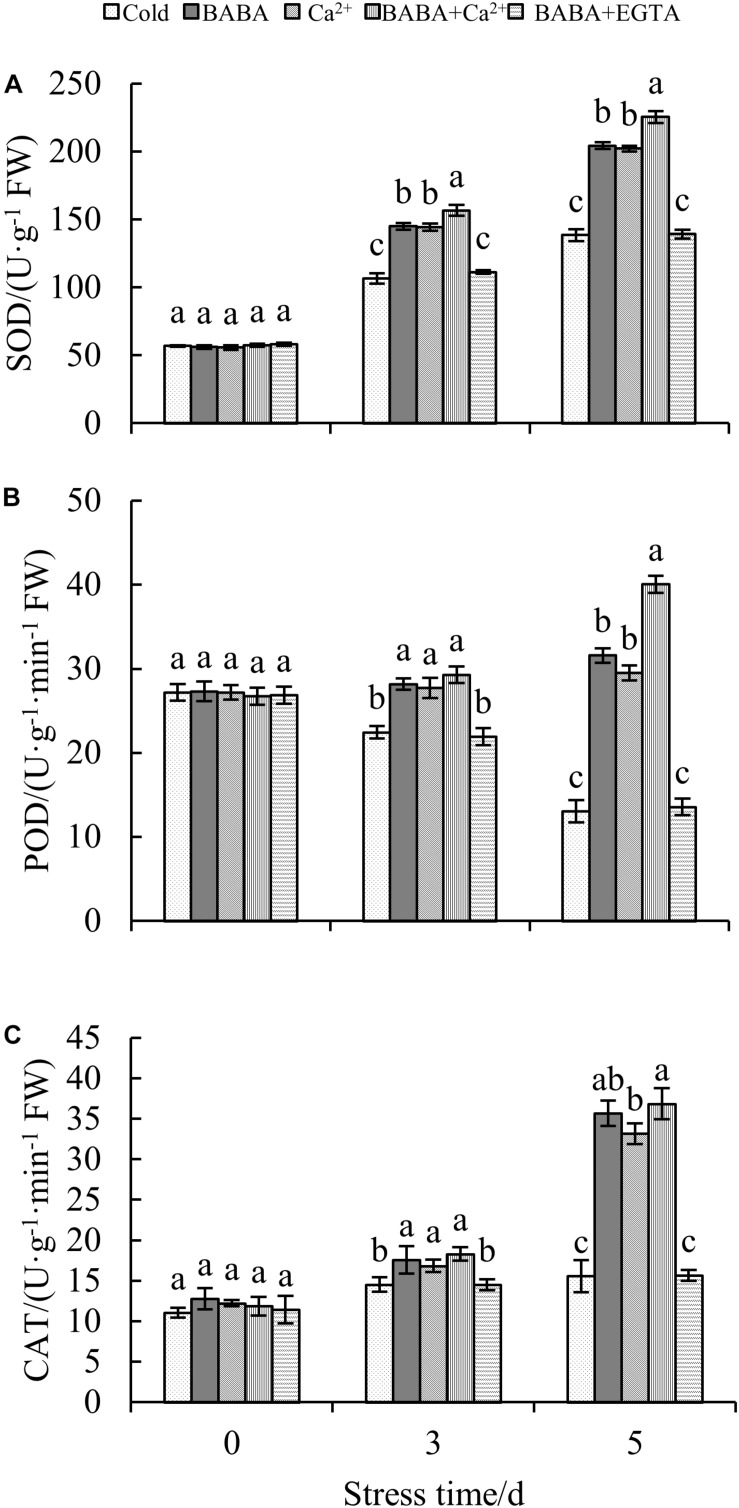
Effects of BABA, Ca^2+^, BABA + Ca^2+^, and BABA + EGTA on SOD **(A)**, POD **(B)**, and CAT **(C)** of tobacco under chilling stress. Data is the mean value ± SE from three replicates. The values with the same letter are not significantly different at *P* < 0.05 according to Duncan’s test.

In addition to the non-enzymatic antioxidant system, the antioxidant enzyme system is also important for plant resistance to oxidative stress. To investigate the mitigative effect of BABA + Ca^2+^ on antioxidant enzymes in tobacco seedlings exposed to cold stress, CAT, POD, and SOD were selected and their activities were determined. Increases in both SOD and CAT activities were clearly observed with prolonged cold treatment. In particular, BABA + Ca^2+^ treatment resulted in peak SOD and CAT activities among the five treatments (Figures 5A,C). When exposed to 5 days of chilling stress, BABA, Ca^2+^, BABA + Ca^2+^, BABA + EGTA, increased SOD activity by 47.55, 46.01, 62.88, and 0.59%, respectively, compared to that in the 5-day cold treatment control group. CAT activity under the four treatments was increased or changed by 1. 29-, 1. 13-, and 1.37-fold and 0.71%, respectively, compared to that in the 5-day cold control group. However, the trend in POD activity was different from that of SOD and CAT activities over time, and BABA + Ca^2+^ resulted in maximum POD activities on day 5 (Figure 5B). Overall, as chilling time was extended, there were remarkable increases in SOD and CAT activities but a decrease in POD activities. Nevertheless, BABA + Ca^2+^ alleviated chilling injury markedly in tobacco leaves and BABA + EGTA reversed the effect on chilling-treated plants, which was consistent with the change in non-enzyme antioxidant contents.

### Combing BABA and Ca^2+^ Increases Hormone Contents in Tobacco Leaves

**FIGURE 6:**
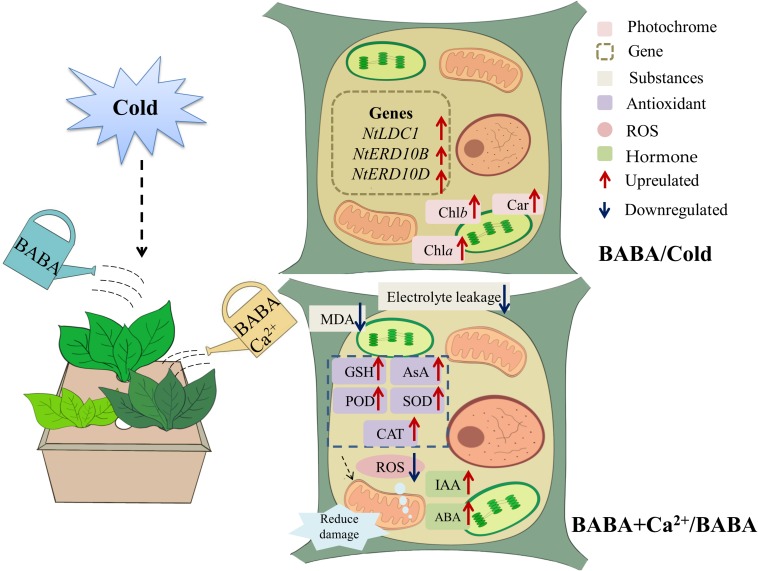
Schematic representation of mechanism through which BABA or BABA + Ca^2+^ protects tobacco from chilling.

IAA is an important intrinsic regulator of cell growth. Compared to that with cold treatment, IAA content with BABA, Ca^2+^, and BABA + Ca^2+^ treatments was increased by 1. 79-, 1. 89-, and 3.86-fold, respectively (Table 4). BABA + EGTA treatment only increased this by 64.28% compared to levels with cold treatment only. The phytohormone ABA is crucial to modulate the response of plants to adverse environments. BABA, Ca^2+^, and BABA + Ca^2+^ improved the ABA content by 1. 79-, 1. 89-, and 3.86-fold compared to levels with cold treatment only. These findings could indicate that BABA and Ca^2+^ could partly modulate plant hormones to accommodate chilling conditions, and that BABA + Ca^2+^ results in even better regulatory effects (Figure 6).

**TABLE 4:** Effects of BABA, Ca^2+^, BABA + Ca^2+^, and BABA + EGTA on IAA and ABA content of tobacco seedlings under cold stress.

Treatment	IAA (μg g^–1^ FW)	ABA (μg g^–1^ FW)
Cold	0.28 ± 0.03 e	0.91 ± 0.01 d
BABA	0.78 ± 0.02 c	1.30 ± 0.02 b
Ca^2+^	0.81 ± 0.01 b	1.29 ± 0.01 b
BABA + Ca^2+^	1.36 ± 0.01 a	1.62 ± 0.02 a
BABA + EGTA	0.46 ± 0.01 d	0.95 ± 0.01 c

*The data are means ± SE, n = 3. Different letters in the same column contend a significant difference between treatments at P < 0.05.*

## Discussion

Chilling stress is a widespread factor that adversely affects plant growth. In crops adapted to high-temperature growth conditions, low temperature is an important factor affecting yield and quality. Tobacco is a cold-sensitive plant, which also serves as a good model to study plant chilling damage. Several studies agree that BABA can alleviate different biological and abiotic stresses including *Colletotrichum orbiculare*, water stress, gray mold, and *Phytophthora infestans*, among others (Jeun et al., 2007; Macarisin et al., 2009; Maymoune et al., 2015; Wang et al., 2015; Kuźnicki et al., 2019). However, less information is available about the effect of BABA on plant defense against low temperatures. In the present study, our data showed that 0.2 mM BABA could effectively relieve chilling damage to tobacco leaves by protecting the cell structure, promoting photosynthetic pigment synthesis, and upregulating stress-related gene expression. Furthermore, a combination of BABA and Ca^2+^ resulted in higher tobacco resistance to chilling. The related mechanisms were found to involve enhancing the antioxidant system, maintaining membrane integrity, and regulating phytohormone content.

The chilling condition is an abiotic stress that induces cell dehydration, resulting in decreased membrane fluidity and a disruption in ion homeostasis in plants. Tobacco, a cold-sensitive model plant, undergoes a series of intricate physiological and biochemical changes after being subjected to chilling stress, including the induction of temperature response signals, modulation of gene expression, and alterations to the membrane structure and metabolic pathways. As is widely accepted, plant morphology and biomass are intuitive responses to plant growth. It has been reported that silicon promotes winter wheat growth under cold conditions by increasing the dry weight and water content of plants (Liang et al., 2008). The research findings suggested that the resistance of tobacco seedlings to chilling stress was promoted mainly by PAs (Wang C. et al., 2019; Wang Y. et al., 2019). Our data found that during tobacco seedling growth, cold stress reduces root length, leaf length, leaf width, and leaf area, but that exogenous BABA application could improve these morphological indexes markedly.

It is well known that photosynthesis is a vital physiological process in plant growth and is highly sensitive to temperature changes. Photosynthetic pigment content is directly related to photosynthesis, which also is widely used to reflect leaf damage. Increasing photosynthetic pigment content is beneficial to enhance photosynthesis. In the present study, BABA application promoted the synthesis of photosynthetic pigments in tobacco seedlings grown at a low temperature. Under these conditions, carotenoids not only act as photoreceptors for the transport of photosynthetic electrons but also function as scavengers of excess ROS, which are generated during stress (Dall’Osto et al., 2007; Havaux et al., 2007). Chloroplast thylakoid membrane permeability is also increased and results in morphological changes under low temperature. Then, the contents of Chl *a*, Chl *b*, and Car are decreased, and the lipid membranes of the photosynthetic organs are damaged, ultimately destroying the photosynthetic system (Wu et al., 2014). Our results showed that 0.2 mM BABA could maintain the cell ultrastructure and increase the content of Chl *a*, Chl *b*, and Car significantly, indicating that it could strengthen tobacco cold tolerance by protecting the photosynthetic system.

Previous studies have shown that BABA regulates gene expression under abiotic stress (Hossain et al., 2012). We further selected three stress-related genes to explore the effect of BABA on resistance-associated gene expression. qPCR analysis showed that 0.2 mM BABA could upregulate *NtLDC1*, *NtERD10B*, and *NtERD10D*. Kasuga et al. (2004) reported that *NtERD10B* and *NtERD10D* contain a promoter region with a DER/CRT element shared by abiotic stress-related genes and that the expression of this element could increase tobacco resistance. Moreover, the *NtERD10B* and *NtERD10D* genes are regulated by the transcription factor DREBIA, for which expression levels represent the strength of stress resistance (Sarmah et al., 2012). Lysine decarboxylase (LDC) is a key enzyme involved in alkaloid synthesis and also has detoxifying effects. The main biological function of LDC is to catalyze the decarboxylation of lysine to produce cadaverine (CAD), and the content of CAD and LDC is related to the resistance of plants (Kim et al., 2006; Kuznetsov et al., 2007). Previous studies have shown that the cold tolerance of plants is positively correlated with the expression of LDC genes. In cucumber plants, LDC gene expression was found to be improved in cold-tolerant varieties, whereas cold-sensitive varieties could not induce LDC gene expression (Lu et al., 2005; Miao et al., 2013). In tobacco plants, Zhang et al. (2016) showed that the *NtLDC1* gene plays a crucial role in the response of tobacco to cold stress and that the cold tolerance of tobacco is increased by upregulated *NtLDC1* gene expression. Consistently, our study also found that exogenous BABA application upregulated the expression of the *NtLDC1* gene in tobacco, which contributed to the enhanced tolerance of tobacco against chilling stress. Therefore, the upregulated expression of these three resistance genes could indicate that BABA improves tobacco cold resistance. Nevertheless, further work, such as overexpression or silencing of the LDC gene in tobacco, should be conducted to obtain more information.

It is widely accepted that the Ca^2+^ signaling pathway is the core upstream signaling pathway and that Ca^2+^ could improve the cold tolerance of plants including tobacco. Therefore, the utilization of Ca^2+^, BABA, and EGTA could be useful for studies on the signaling pathway of BABA with respect to relieving cold stress. As mentioned, we combined BABA with Ca^2+^ and found that the combined effect was better than that with either alone. The ability of BABA + EGTA treatment to alleviate cold stress suggested that EGTA could block the Ca^2+^ signaling pathway under cold conditions, and it also shows that the effect of BABA on alleviating chilling stress is closely related to the calcium signaling pathway. This result is consistent with research in other plant species, including *Stylosanthes guianensis* (Zhou and Guo, 2009) bermudagrass (Shi et al., 2014), and *Camellia sinensis* (Ding et al., 2019). Previous research substantiates the belief that cold stress induces oxidative stress and destroys the cell membrane structure (Gill and Tuteja, 2010). MDA content and electrolyte leakage are important indicators closely related to cold resistance. It has been documented that cell damage causes tissue dehydration, accompanied by an increase in MDA content and electrolyte permeation (Ding et al., 2017). In this study, we demonstrated that the exogenous administration of BABA, CaCl_2_, and BABA + Ca^2+^ could reduce electrolyte penetration and MDA content. Thus, we suggest that BABA + Ca^2+^ can reverse the deleterious effects of cold stress to a greater extent. Furthermore, oxidative damage will also lead to changes in antioxidant enzyme system activity and ROS levels. Several studies have reported that cold stress triggers an increase in ROS content in plant cells. Excessive ROS accumulation destroys organelles and disrupts membrane function and structural integrity (Apel and Hirt, 2004; Asada, 2006). However, plants ease this membrane damage due to superabundant ROS accumulation through antioxidant systems including enzymatic and non-enzymatic protective mechanisms (Zhou et al., 2012). The former contains SOD, POD, and CAT, whereas the latter includes AsA and GSH. In general, as low temperature is maintained, the reaction rate of antioxidant enzymes lags behind the rate of ROS accumulation (Kamrul et al., 2014; Hu et al., 2016). Results revealed that treatments of BABA, Ca^2+^, and BABA+Ca^2+^ could reduce ROS accumulation, along with elevated antioxidant content and increased antioxidant enzyme activities. However, the ROS accumulation with BABA + EDTA treatment was not significantly different from that with cold treatment. The first line of the plant defense system involves the conversion of excess superoxide ions to H_2_O_2_, catalyzed by SOD, which in turn causes POD and CAT to convert H_2_O_2_ to H_2_O and O_2_^∙⁣–^. Itcould be extrapolated that BABA coordinates three antioxidant enzymes and non-enzymatic antioxidants such as GSH and AsA to optimize the elimination of ROS, which is closely related to the calcium ion pathway. This is consistent with the research of Mostek et al. (2016), showing that BABA increases salt tolerance in barley seeds by upregulating antioxidant enzymes, PR proteins, and molecular chaperones. Similarly, Li et al. (2018) determined that melatonin can alleviate cold injury in camellia plants by improving GSH content. From this, we considered that the mechanism of BABA antioxidant defense is closely related to the Ca^2+^ signal and that a combination of BABA and Ca^2+^ could further enhance plants antioxidant defense, thereby improving the resistance of tobacco to chilling stress.

Plant hormones play an important role in the cold adaptive processes. The signaling pathways induced by exogenous substances in plants are related to the hormone network that ultimately regulates plant stress responses under chilling stress (Pastor et al., 2013). BABA activates the jasmonic acid signal transduction pathway via the antioxidant defense system and includes stomatal closure, which contributes to improved plant drought resistance (Du et al., 2012). In addition to cold, drought, and salt stress, when plants are attacked by pathogens, BABA binds receptor proteins in the plant pathogen system, thereby activating ABA, salicylic acid, and JA signaling pathways (Zimmerli et al., 2000; Ton and Mauch-Mani, 2004). Thus, upon exposure to biotic and abiotic stresses, BABA-mediated responses might be based on the activation of ABA-dependent signaling. One study showed that ROS signaling might interact with ABA to confer cold stress tolerance in certain species (Megha et al., 2017). IAA and ABA play a substantial role in the regulation of plant growth and development under cold stress. In this study, we verified that BABA, Ca^2+^, and BABA + Ca^2+^ treatment could significantly upregulate ABA and IAA in tobacco exposed to low temperature. However, EGTA reversed the effect of BABA on chilling-treated plants, which is consistent with results of our previous studies.

## Conclusion

Chilling temperatures are responsible for a series of physiological disturbances in tobacco. The results of this study showed the following: (1) BABA could relieve cold injury in tobacco and the optimum concentration proved to be 0.2 mM; (2) BABA is beneficial to maintain the cell structure and upregulate stress-related gene expression; (3) combination of BABA and Ca^2+^ results in a better mitigative effect on chilling stress; (4) the enhancement of cold tolerance in tobacco mediated by BABA is closely related to the state of Ca^2+^ signaling. This study presented a feasible and eco-friendly method to improve plant resistance against chilling stress. Nevertheless, more detailed mechanism of BABA and Ca^2+^ should be expounded in plants and the application of BABA and Ca^2+^ in field experiments needs to be examined.

## Data Availability Statement

All datasets generated for this study are included in the article/Supplementary Material.

## Author Contributions

X-HM and Z-CX conceived the idea and designed the research. X-HM, J-YX and WJ performed the experiments and analyzed the data. Z-CX and WJ supervised the study. DH, W-XH and B-JD made a modification of the manuscript. X-HM wrote the manuscript with contributions from other co-authors. All authors contributed to the manuscript revision, and read and approved the submitted version.

## Conflict of Interest

The authors declare that the research was conducted in the absence of any commercial or financial relationships that could be construed as a potential conflict of interest.

## Footnotes

1 www.ncbi.nlm.nih.gov
