# Supplementary material for: Combination of β-Aminobutyric Acid and Ca2+ Alleviates Chilling Stress in Tobacco (Nicotiana tabacum L.)
Source: Front Plant Sci. 2020 May 13;11:556. doi: 10.3389/fpls.2020.00556 (PMC7237732; doi:10.3389/fpls.2020.00556)
Supplement: Supplementary file 1 [file Table_1.DOCX]

Supplementary Material

# Supplementary Note

From the related research, three low temperature groups (4/0°C, 8/4°C, 12/8°C) and seven days of stress imparting time were used to study plant cold tolerance (Hodges et al., 2006; Balabusta et al., 2016; Diao et al., 2017; Yang et al., 2018). A pre-experiment was carried out to define the experiment temperature and stress imparting days. Normal temperature (28/18°C) served as the control, and three replicates were performed for each treatment. Tobacco leaves treated with 4/0°C group exhibited withered and died on the 3rd day. Compared with normal temperature, significant differences of tobacco leaves were detected under 8/4°C and 12/8°C treatment from the 3rd day. And, the the state of plant growth tended to be stable after the 5th day. Furthermore, 8/4°C treatment seemed to causes more obviously change from the normal growth. Thus, 8/4°C was selected and 0, 3 and 5 stress imparting days were used in this study. The related results were served as supplementary material.

# Supplementary Table

**Supplementary Table 1.** Morphological parameters of tobacco treated with different low temperatures

| Treatment | | 0 d | 1 d | 2 d | 3 d | 4 d | 5 d | 6 d | 7 d |
| --- | --- | --- | --- | --- | --- | --- | --- | --- | --- |
| Leaf length(cm) | 28/18°C | 16.87±0.15 a | 17.10±0.10 a | 17.47±0.31 a | 18.00±0.40 a | 18.30±0.30 a | 19.00±0.26 a | 20.20±0.20 a | 22.50±0.20 a |
|  | 4/0°C | 16.97±0.15 a | 16.97±0.32 a | 16.90±0.36 b |  |  |  |  |  |
|  | 8/4°C | 16.93±0.25 a | 17.13±0.21 a | 17.33±0.25 ab | 17.60±0.20 a | 17.67±0.15 c | 17.87±0.25 c | 17.93±0.31 c | 17.90±0.36 c |
|  | 12/8°C | 16.97±0.21 a | 17.27±0.12 a | 17.57±0.15 a | 17.83±0.21 a | 18.07±0.32 b | 18.43±0.21 b | 18.57±0.15 b | 18.60±0.10 b |
| Leaf width(cm) | 28/18°C | 6.13±0.15 a | 6.50±0.10 a | 6.97±0.12 a | 7.43±0.15 a | 7.90±0.10 a | 8.67±0.06 a | 8.93±0.21 a | 10.53±0.25 a |
|  | 4/0°C | 6.20±0.20 a | 6.23±0.15 a | 6.10±0.17 c |  |  |  |  |  |
|  | 8/4°C | 6.13±0.15 a | 6.40±0.20 a | 6.53±0.21 b | 6.80±0.10 b | 7.10±0.20 b | 7.90±0.26 b | 8.07±0.21 b | 8.10±0.20 b |
|  | 12/8°C | 6.10±0.20 a | 6.40±0.20 a | 6.83±0.21 ab | 7.20±0.20 a | 7.57±0.25 a | 8.13±0.15 b | 8.27±0.21 b | 8.37±0.15 b |
| Leaf area(cm^2^) | 28/18°C | 65.64±1.99 a | 70.52±0.95 a | 77.21±1.66 a | 84.89±2.10 a | 91.74±2.66 a | 104.48±1.13 a | 114.48±2.03 a | 150.39±4.54 a |
|  | 4/0°C | 66.74±1.81 a | 67.09±0.79 a | 65.43±3.03 b |  |  |  |  |  |
|  | 8/4°C | 65.91±2.63 a | 69.59±2.99 a | 71.87±3.27 a | 75.94±1.73 b | 79.58±1.86 b | 89.53±2.06 c | 91.77±1.34 c | 91.98±1.66 c |
|  | 12/8°C | 65.67±2.46 a | 70.12±2.23 a | 76.18±2.97 a | 81.49±3.18 a | 86.77±4.38 a | 95.14±2.79 b | 97.40±3.19 b | 98.75±2.32 b |

The data are means ± SE, n = 3. Bars with different letters are significantly different at the 0.05 level (Duncan’s test).

# Supplementary Reference

Balabusta, M., Katarzyna, S., and Malgorzata., M.P. (2016). Exogenous melatonin improves antioxidant defense in cucumber seeds (*Cucumis sativus* L.) Germinated under chilling stress. *Front Plant Sci.* 7, 575. doi: 10.3389 / fpls.2016.00575

Diao, Q., Song, Y., Shi, D., and Qi, H. (2017). Interaction of polyamines, abscisic acid, nitric oxide, and hydrogen peroxide under chilling stress in tomato (*Lycopersicon esculentum* Mill.) seedlings. *Front Plant Sci.* 8, 203. doi:10.3389/fpls.2017.00203

Hodges, D.M., Andrews, C.J., Johnson, D.A., and Hamilton, R.I. (2006). Antioxidant compound responses to chilling stress in differentially sensitive inbred maize lines. *Physiol. Plantarum* 98, 685-692. doi: 10.1034/j.1399-3054.1996.980402.x

Yang, Y.J., Zhang, S.B., and Huang, W. (2018). Chloroplastic ATP synthase alleviates photoinhibition of photosystem I in tobacco illuminated at chilling temperature. *Front Plant Sci.* 14, 1648. doi: 10.3389/fpls.2018.01648
